# Male involvement in prevention programs of mother to child transmission of HIV: a systematic review to identify barriers and facilitators

**DOI:** 10.1186/2046-4053-2-5

**Published:** 2013-01-16

**Authors:** Frederick Morfaw, Lawrence Mbuagbaw, Lehana Thabane, Clarissa Rodrigues, Ana-Paula Wunderlich, Philip Nana, John Kunda

**Affiliations:** 1 Department of Obstetrics and Gynaecology, Faculty of Medicines and Biomedical Sciences, University of Yaounde 1, P.O Box 1364, Yaounde, Cameroon; 2 Centre for the Development of Best Practices in Health (CDBPH), Yaoundé Central Hospital, Henri Dunant Avenue, Messa, PO Box 87, Yaoundé, Cameroon; 3 Departments of Clinical Epidemiology and Biostatistics, Pediatrics and Anesthesia, McMaster University, Hamilton, ON, Canada; 4 Biostatistics Unit, Father Sean O’Sullivan Research Centre, St Joseph’s Healthcare, Hamilton, ON, Canada; 5 Research and Innovation Coaching Program, Department of Surgery at Duke University, Durham, North Carolina, USA; 6 Faculty of Inga, Maringa State University, Maringa Area, Brazil; 7 Community Information and Epidemiological Technologies (CIET) Zambia, Lusaka, Zambia

**Keywords:** Male, Partner, Involvement, HIV, PMTCT, Barriers, Facilitators

## Abstract

**Background:**

Many reports point to the beneficial effect of male partner involvement in programs for the prevention of mother-to-child-transmission (PMTCT) of HIV in curbing pediatric HIV infections. This paper summarizes the barriers and facilitators of male involvement in prevention programs of mother-to-child-transmission of HIV.

**Methods:**

We searched PubMed, EMBASE, CINAHL and the Cochrane Central Register of Controlled Trials (CENTRAL) for studies published in English from 1998 to March 2012. We included studies conducted in a context of antenatal care or PMTCT of HIV reporting male actions that affected female uptake of PMTCT services. We did not target any specific interventions for this review.

**Results:**

We identified 24 studies from peer-reviewed journals; 21 from sub-Saharan Africa, 2 from Asia and 1 from Europe. Barriers to male PMTCT involvement were mainly at the level of the society, the health system and the individual. The most pertinent was the societal perception of antenatal care and PMTCT as a woman’s activity, and it was unacceptable for men to be involved. Health system factors such as long waiting times at the antenatal care clinic and the male unfriendliness of PMTCT services were also identified. The lack of communication within the couple, the reluctance of men to learn their HIV status, the misconception by men that their spouse’s HIV status was a proxy of theirs, and the unwillingness of women to get their partners involved due to fear of domestic violence, stigmatization or divorce were among the individual factors.

Actions shown to facilitate male PMTCT involvement were either health system actions or factors directly tied to the individuals. Inviting men to the hospital for voluntary counseling and HIV testing and offering of PMTCT services to men at sites other than antenatal care were key health system facilitators. Prior knowledge of HIV and prior male HIV testing facilitated their involvement. Financial dependence of women was key to facilitating spousal involvement.

**Conclusions:**

There is need for health system amendments and context-specific adaptations of public policy on PMTCT services to break down the barriers to and facilitate male PMTCT involvement.

**Trial Registration:**

The protocol for this review was registered with the International prospective register of systematic reviews (PROSPERO) record CRD42011001703.

## Background

By 2009, a total of 33.3 million people worldwide were living with the human immunodeficiency virus (HIV), 2.5 million of whom were children < 15 years [1]. Two-thirds of the total number of people living with HIV worldwide were from sub-Saharan Africa, 2.3 million of which were children < 15 years [1]. In 2009, there were a total of 1.8 million HIV-related deaths, 260,000 of these being children <15 years [1]. Again, sub-Saharan Africa accounted for 72.2% (1.3 million) of these deaths and a total of 88% of the deaths in children <15 years [1]. A total of 370,000 children were estimated to be infected with HIV through mother-to-child transmission (MTCT) in 2009 [1]. These figures indicate not only the magnitude of the problem, but also the fact that pediatric HIV infections are numerous and worrisome. They highlight a need to step up the current efforts at preventing pediatric HIV infections.

Programs for the prevention of mother-to-child-transmission (PMTCT) of HIV are part of the solution to eliminate new pediatric HIV infections. Their primary goal is to prevent the transmission of HIV from an HIV-positive mother to her baby [2]. They represent the third prong of the World Health Organization’s (WHO) four-pronged approach [3] to prevent HIV infections in mothers and infants. PMTCT interventions can be summarized as maternal antenatal HIV testing, the uptake of prophylactic antiretroviral therapy, and formula feeding or exclusive breastfeeding for HIV-infected women [4]. However, adequate uptake and adherence to these PMTCT interventions have been challenging for some women if their partners are unaware of or do not support their involvement [5]. The reality is that traditional PMTCT programs focus mostly on women, ignoring the important role of men [6]. Hence, the moderate levels of PMTCT success can be partially explained by this narrow focus on women alone.

The uptake of PMTCT interventions relies on complex decision-making dynamics in some cases [7]. Shared decision-making facilitates participation in health care programs including PMTCT [8,9]. This is most relevant in sub-Saharan Africa where reproductive health decisions are greatly influenced by male partners [10]. Traditionally, male partners tend to have an upper hand in sexual and reproductive health decision-making [11]. They also play a role in reducing female HIV acquisition during pregnancy [12]. Many reports point to the beneficial effect of male partner involvement in antenatal HIV services on prevention of pediatric infections [13-17]. Aluisio *et al.*[14] went as far as providing biological evidence of the benefits of male partner involvement with PMTCT services in preventing mother-to-child transmission of HIV in East Africa.

Since male partners influence women’s ability and willingness to adhere to diverse product use [4], including PMTCT interventions, there is the need for cooperation and agreement between couples to prevent both horizontal and vertical transmission of HIV [13,18]. The lack of male involvement in PMTCT consequently undermines the potential benefits of antenatal HIV preventive efforts [19], thus representing a missed opportunity to effectively prevent vertical HIV transmission. Male partners therefore seem to be the forgotten half of the equation [20].

Despite the potential benefits, male PMTCT involvement has some possible drawbacks. These include disruptions of family relationships, emotional and physical abuse for the spouses, loss of economic support, and blame and abandonment for the spouses [5-21]. Njunga et al. described a situation in Malawi, a setting with a dominant matrilineal system, whereby the PMTCT program was referred to as the ‘divorce program’ as the request for partner disclosure by the program led to numerous family dissolutions [21]. More recently, Kim et al., still in Malawi, demonstrated that women without an involved partner were most likely to complete the PMTCT cascade, while those with involved but undisclosed partners were least likely to complete this cascade [22].

Still the results of several studies indicate that male participation in the PMTCT program needs to be enhanced [14,23-25]. Increased male involvement was even been identified as a critical strategy for the President’s Emergency Plan for AIDS Relief (PEPFAR) countries to enhance implementation of PMTCT interventions [26].

Yet achieving male involvement in communities where their involvement is assessed to be potentially beneficial can be challenging for health systems worldwide. Efforts made to involve men in antenatal voluntary counseling and testing have achieved limited successes [23-25]. This is because evidence-based strategies for effectively engaging men in women’s health are limited [4]. A potential way forward will be to identify the facilitators and barriers for male partner involvement in PMTCT to improve the uptake and effectiveness of the program.

This review seeks to identify the facilitators of and barriers to male involvement in PMTCT activities in order to inform programs aimed at enhancing male partner participation in PMTCT. This report is part of a larger systematic review of the evidence intended to determine the effect of male participation on female uptake of PMTCT services to evaluate the proportion of men who accept voluntary counseling and testing for HIV, provide moral and financial support to their spouses to adhere to antenatal care and PMTCT guidelines, etc. [27]. For the purpose of this review, male partner involvement is understood as a broad and multifaceted concept including all forms of male partner support for women’s participation in PMTCT programs, including but not limited to financial or moral support, actual physical presence and accepting HIV testing. Being a systematic review of published literature with no interventions on humans or animals, we did not need ethical approval in order to carry out the review. However, each of the studies included in the review received appropriate ethical approval from their respective ethical review boards.

### Objectives

The objective of this review is to determine the barriers to and facilitators of male partner involvement in PMTCT activities.

## Methods

### Protocol and registration

The methods for this systematic review have been published in detail elsewhere [27]. The protocol for this review was registered with the International prospective register of systematic reviews (PROSPERO) record CRD42011001703 [28].

### Overview of methods

We did a comprehensive electronic search of four electronic databases: PubMed, EMBASE, CINAHL and the Cochrane Central Register of Controlled Trials (CENTRAL). We limited ourselves to studies published in English from 1998 to March 2012. The following search terms and their MeSH (medical subject heading) equivalents were used in varying combinations to search the different databases: HIV, male, spouse, partner, men, couple, pregnancy, gestation, participation, involvement, engagement, antenatal, barriers, facilitators, disclose, declare, testing, PMTCT, voluntary counseling and testing (VCT), vertical transmission, domestic violence. The search strategy was reviewed by the Research on Research (RoR) team of Duke University, Durham, USA [29]. We improved upon the sensitivity of our searches by applying keywords, and reviewing bibliographies of eligible studies identified early in the review process. The last search was run on the 19^th^ March 2012. We included randomised controlled trials (RCTs) and observational studies conducted in a context of antenatal care (ANC) or prevention services of mother-to-child transmission (PMTCT) of HIV reporting male actions that affected female uptake of PMTCT services. The study participants were men, women, or focus groups of the community. No specific interventions were targeted for this review.

After screening for duplicates, abstracts of potentially eligible studies were reviewed. The full texts of eligible studies were retrieved and their methodological quality assessed using the Jadad scale [30] for RCTs, and the Newcastle-Ottawa scale (NOS) [31] for observational studies. A pretested data abstraction form was used to abstract data from the included studies. In most of the studies, data were collected using interviews. In four studies, data were collected from patient’s records [32-35]. In some cases, barriers or facilitators were not the main focus of the studies, but were either identified in the results or discussion sections. The Kappa statistic was used to assess agreement between the data abstractors (FM and LM) at each stage. Discrepancies were sorted out by discussion or consultation with a third party (LT).

## Results

### Results of the search

**Figure 1:**
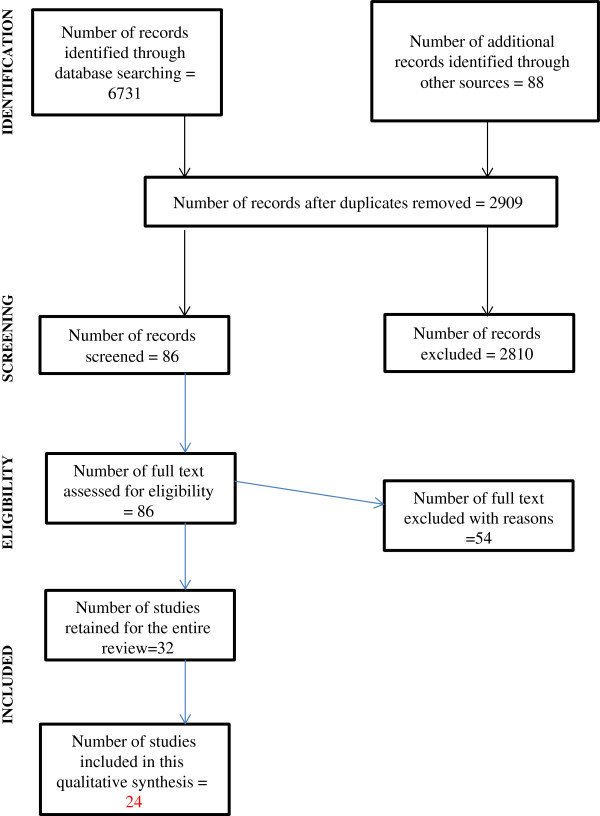
PRISMA diagram for the flow of information through different phases of the review.

The PRISMA diagram in Figure 1 summarises the selection process at each stage.

Thirty-two studies were retained for data extraction, of which 24 reported data needed in this qualitative report (see Table 1). There was some disagreement between the two authors on studies to be included (Kappa estimate 0.57, 95% CI 0.41, 0.74; p<0.001). This disagreement was resolved by discussion. The Kappa estimate for agreement on the methodological quality of included studies was 0.84, 95% CI 0.74-0.94; p<0.001. The discrepancies were also sorted out by discussion with the third author. The quality scores are reported in Table 1.

**Table 1:** Characteristics of included articles

**PAPER (reference)**	**COUNTRY/ SETTING**	**STUDY DESIGN**	**STUDY POPULATION**	**MAIN STUDY OBJECTIVE(S)**	**QUALITY SCORE**
Aarnio et al 2009 [19].	Malawi, Rural.	Cross sectional/mixed method.	388 men and 11 focus groups.	To explore men’s perception on HIV in pregnancy and their involvement in antenatal VCT.	4/4
Aluisio et al 2011 [14].	Kenya, Urban.	Prospective cohort.	510 HIV infected pregnant women.	To investigate the relationship between male involvement in PMTCT and infant HIV acquisition/ mortality.	7/9
Becker et al 2010 [38].	Tanzania, Urban.	RCT.	1521 pregnant women.	To evaluate the acceptance/ effectiveness of couple VCT relative to individual VCT in ANC.	2/5
Byamugisha et al 2010 [34].	Uganda, Urban.	Case control.	54429 pregnant ANC attendees.	To describe the first seven years of the PMTCT programme.	6/8
Byamugisha et al 2011 [36].	Uganda, Urban.	RCT.	1060 pregnant women.	To evaluate the effect of an invitation letter to spouses on couple attendance and partner acceptance of HIV testing.	5/5
Desgrees-Du-Lou et al 2009a [42].	Cote d’Ivoire, Urban.	Cohort.	937 pregnant women.	To determine the effect of prenatal VCT on couple communication about STIs, HIV and sexual risk prevention.	6/9
Ditekemena et al 2011 [45]	Democratic Republic of Congo, Urban.	RCT.	2706 pregnant women.	1) To identify alternative strategies to increase male participation in VCT 2) To explore factors associated with male/couple participation in VCT.	3/5
Falnes et al 2011 [39]	Tanzania, Urban and Rural.	Cross sectional/ Mixed method.	426 postpartum women bringing their children for the first dose of vaccine plus 9 FGD.	To explore the acceptability of the PMTCT programme and identify structural/ cultural challenges to male involvement.	4/4
Farquhar et al 2001 [15]	Kenya, Urban.	Cohort.	172 HIV seropositive pregnant women.	To determine the association between partner notification and infant feeding decisions.	7/9
Farquhar et al 2004 [25]	Kenya, Urban.	Cohort.	2836 pregnant women	To assess the impact of partner involvement, on perinatal intervention uptake and condom use.	8/9
Homsy et al 2006 [23]	Uganda, Rural.	Cross sectional.	3591 pregnant women accepting HIV counselling and testing.	To determine the acceptability, feasibility and uptake of intrapartum HIV counseling and testing and PMTCT services by women, men and couples.	4/4
Jasseron et al 2011 [35]	France, Urban and rural.	Cohort.	2952 pregnant women.	To identify the proportion non-disclosure of HIV, and the associated factors, and the impact of non-disclosure on PMTCT uptake.	7/9
Kakimoto et al 2007 [33]	Cambodia, Urban.	Case control.	20757 pregnant ANC attendees during the study period.	To evaluate the influence of partner participation in the mother class to PMTCT services.	6/8
Katz et al 2009a [47]	Kenya, Urban.	Cohort.	313 men accompanying their spouses to ANC.	To understand male non-disclosure of HIV status in antenatal care.	6/9
Katz et al 2009b [48]	Kenya, Urban.	Cohort.	313 men accompanying their spouses at ANC.	To identify methods to increase male involvement in antenatal VCT.	5/9
Kiarie et al 2006 [16]	Kenya, Rural.	Prospective cohort.	2836 pregnant women attending antenatal care.	To determine the impact of domestic violence on uptake of interventions to PMTCT.	7/9
Kizito et al, 2008 [32]	Uganda, Urban.	Case control.	20738 pregnant ANC attendees.	To describe uptake of HIV and syphilis testing in a PMTCT programme.	6/8
Mbonye et al 2010 [37]	Uganda, Rural.	Cross sectional.	10706 women age 14-49 years.	To explore perceptions, care-seeking practices and barriers to PMTCT among young HIV positive women.	4/4
Mohlala et al 2011 [20]	South Africa, Urban.	RCT.	1000 pregnant women with gestational age less than 30 weeks	To assess the acceptability/ feasibility of pregnant women inviting their male partners to ANC and VCT	3/5
Msuya et al 2008 [40]	Tanzania, Urban.	Cohort.	2654 pregnant women in the third trimester of pregnancy.	Prevalence and predictors of male partner participation in HIV VCT, and the effect of partner participation on uptake of PMTCT interventions.	6/9
Nkuoh et al 2010 [44]	Cameroon, Rural.	Cross sectional.	252 men completed the survey.	To identify barriers to men participating in their wives’ ANC and obtaining HIV testing.	2/4
Shankar et al 2003 [46]	India, Urban.	Cross sectional/mixed method.	144 women, 100 men, 15 in-depth interviews.	To determine the acceptability for HIV testing within ANC and delivery room.	3/4
Theuring et al 2009 [41]	Tanzania, Urban.	Cross sectional mixed method.	124 men and 6 FGDs.	To learn about men’s perspectives on and the experiences with ANC.	3/4
Tonwe-Gold et al 2009 [43]	Cote d'Ivoire, Urban.	Cross sectional.	605 HIV infected pregnant women.	To describe a family focused approach to HIV care and treatment.	6/9

ANC= Antenatal care; VCT= Voluntary counselling and testing for HIV; RCT= Randomized controlled trial; HIV=Human Immuno-deficiency virus; PMTCT= Prevention of mother-to-child-transmission; FGD=Focus group discussion; STI= Sexually Transmitted Infection.

The excluded studies are listed in Appendix 1.

### Included studies

The studies included were mostly from developing countries. Six of the studies were conducted in Kenya [14–16;25,36,37], five in Uganda [23,32,34,36,37]. Tanzania had four studies [38-41], while Cote d’Ivoire had two [42,43]. The rest of the countries had one study each: Cameroon [44], Malawi [19], Democratic Republic of Congo [45], South Africa [20], India [46], Cambodia [33] and France [35]. The lone study from the developed world was a study conducted in France [35], yet the majority of the study participants were African immigrants. Most of the included studies were observational studies; nine were cohort studies [14-16,25,35,40,42,47,48]; eight were cross sectional studies [19,23,37,39,41,43,44,46]; and three were case control studies [32-34]. Four of the included studies were randomized controlled trials [20,36,38,45].

See Table 1 for the characteristics of the included studies.

### Barriers to male partner involvement in PMTCT

Sixteen of the included studies identified at least one barrier to male partner participation in PMTCT. The reports were numerous and recurrent with over 25 different barriers identified, grouped into seven themes: male individual barriers, female barriers, societal / cultural barriers, health system barriers, information/knowledge barriers, barriers linked to disagreement with PMTCT teachings, and barriers due to relationship dynamics. Table 2 below summarises the main barriers identified. The relative importance of each barrier was based on the number of studies reporting the barrier. This criterion was used rank the different barriers reported in Table 2.

**Table 2:** Summary table of barriers to male PMTCT involvement

**BARRIERS TO MALE PMTCT INVOLVEMENT**	
**Societal/cultural barriers**	
**• **Perception of antenatal care as a woman’s place [19,23,37,40-43,45,46].	
**• **Cultural norm that men should not participate in antenatal care as pregnancy is a woman’s affair [25,37,43,46].	
**• **Societal ridicule of men accompanying their wives to ANC [19,32,46]	
**• **Women are not allowed to lead [41,46].	
**• **Conflict between PMTCT recommendations and cultural norms such as breastfeeding [39].	
**• **Cultural patterns of communication [44]	
**Male individual factors**	
**• **Reluctance to learn ones status [19,32,37,39,43,46].	
**• **Lack of time for ANC/PMTCT [25,37,41,43,46].	
**• **Do not see the benefits of testing [41,43].	
**• **Self-perception of being in good health [32,46].	
**• **Avoidance of the burden of care [19,46].	
**• **Lack of finances [19,46].	
**• **No one left at home to look after the children [44].	
**Information/knowledge barriers**	
**• **Misconception that your partner’s HIV status is a proxy of your own status [37,41,44,46].	
**• **Unawareness of the availability of antenatal VCT by men [19,43,46].	
**• **Men’s limited knowledge of PMTCT [19,43].	
**• **Lack of community awareness on the importance of male PMTCT involvement [25].	
**Health system barriers**	
**• **Long waiting times at the clinic [46,47].	
**• **ANC services are not male-friendly [37,43].	
**• **Distrust in confidentiality of the health system [19].	
**Female factors**	
**• **Women not involving their partners due to numerous fears e.g. accusations of infidelity, divorce, stigmatization, domestic violence [16,25,35,42]	
**Relationship Dynamics**	
**• **Weaker relationships [15,19].	
**• **Fidelity within the relationship [42,46]	
**Relationship Dynamics**	
**• **Weaker relationships [15,19].	
**• **Fidelity within the relationship [42,46]	
**Disagreement with PMTCT teachings**	
**• **Disagreement with PMTCT encouragement of condom use within the couple [39].	
**• **Men perceiving prenatal HIV testing as a late event. They would have been offered the chance to test earlier [40].	

ANC= Antenatal care, PMTCT=Prevention of mother-to-child transmission, VCT=Voluntary counseling and testing, HIV=Human Immuno-deficiency Virus.

#### Societal or cultural barriers

The most frequently reported barrier for male involvement in antenatal care identified across the different studies (38% of the studies) was the perception that antenatal care was a woman’s activity, and it was thus shameful for a man to be found in such settings [19,23,38-41,43,44,48]. This cultural barrier in itself without any other external influence demotivated men from attending antenatal care and getting involved in PMTCT.

In other studies (17% of the studies), it was culture that men should not participate in antenatal care activities [25,41,44,48]. This cultural barrier was independent of antenatal care being perceived as a woman’s place.

This perception was closely linked to the third identified barrier which was the societal ridicule for men who accompanied their wives for ANC, identified in 13% of the studies. These men were ridiculed as being jealous, over-protective of their wives and lacking self-confidence [19,32,44]. This dissuaded men from getting involved.

Another cultural barrier identified was the fact that in most African cultures, women are not allowed to lead [39,44]. In these settings, it is inconceivable for a woman to tell a man what to do, and worse still for him to consent to what she says [39]. This cultural norm forbids women from taking decisions at home on matters such as antenatal care and HIV counselling and testing [39,44] and thus served as a major obstacle to women’s efforts of involving their spouses in PMTCT.

Potential conflict between certain PMTCT recommendations and cultural norms was also found to be barrier. This was especially so with breastfeeding [39]. In a context where mixed infant feeding is the norm, the PMTCT recommendation of non-breastfeeding would go against that norm [39]. Following this PMTCT recommendation was frowned upon by male partners and this created a barrier to respecting the recommendation.

Nkuoh et al. [44] identified cultural communication patterns in which men and women do not fully express themselves, as a barrier to male involvement. This was perpetrated by ‘silence’ on the part of men or ‘non complaint’ on the part of women to give a general impression of all being well. These communication patterns blocked dialogue within couples, thus limiting the chances of male PMTCT involvement.

#### Male individual factor barriers

The reluctance to learn one’s HIV status was a major limiting factor to male ANC/PMTCT involvement, and was reported in 25% of the studies [19,32,37,41,44,48]. This reluctance was grounded in many theories the most common of which was the fear of being HIV positive [32,37,44,48]. For others, this reluctance was associated with the shame of learning one’s HIV status especially if it turned out to be positive [19,37]. Others were reluctant to get involved due to fear of the community stigmatization associated with HIV testing [32]. In some, reluctance was a demonstration of men’s ‘stubborn nature’ [19], while in other cases it was stated that men did not want to participate in ANC/PMTCT activities [48].

The second barrier was the time factor, reported by 21% of the studies [25,39,41,44,48]. The reports noted that the timing of ANC/PMTCT activities was in conflict with men’s normal daily activities. Men simply did not have the time to participate in ANC and receive the knowledge necessary to implement PMTCT strategies.

A further barrier mentioned by 8% of the studies was the perception by men that they simply did not see any benefits in testing for HIV and getting involved in PMTCT [39,41]. Hence the whole exercise was deemed to be futile.

An interesting barrier to male PMTCT involvement was a man’s perception of his own health. This was mentioned in 8% of the studies. Most men deemed themselves to be in good health and this was the major limiting factor for ANC/PMTCT involvement [32,44]. Since HIV testing is the gateway into any PMTCT programme, the self-perception of good health was a limitation to this portal of entry.

The financial barrier was also present and equally stated in 8% of the studies. The lack of finances and consequently the avoidance of the burden of health care hindered men from attending antenatal care and upholding PMTCT recommendations [19,44]. In some cases the men stated that they lacked the money to accompany their partners and pay for health care [44].

The final barrier in this category was the problem of childcare [44]. Nkuoh et al. [44] stated that in the case where both parents were to go for antenatal care, there would be no one left at home to look after the other children.

#### Information/knowledge barriers

The misconception by men that their partner’s HIV status was a proxy for their own status was a major barrier identified in 17% of the studies. The men therefore saw no need to attend PMTCT and get tested provided their partners had been tested for HIV [39,42,44,48].

In general, men were unaware of antenatal voluntary counselling and testing services, a fact highlighted by 13% of the studies [19,41,44]. This barrier was quite pertinent as in the settings where it was described, these men were willing and motivated to get involved in ANC and PMTCT but did not just know where to go for testing. In other settings, despite the fact that men may be motivated to get involved in PMTCT, the lack of the particular information that men were called upon to attend ANC/PMTCT services with their wives was the major limiting factor for their involvement [41].

Also, men had generally limited knowledge on PMTCT [19,41]. They neither understood what it meant nor of what importance it was.

Lack of community awareness on the importance of partner involvement in ANC/PMTCT was also identified a problem [25].

#### Health system barriers

Long waiting times at the antenatal clinic was cited as an obstacle to male involvement in antenatal care in 8% of the studies [44,45]. Other studies found that antenatal health services were perceived as being male unfriendly, and this consequently discouraged men from getting involved [41,48]. Distrust in the confidentiality of the health care system was identified as an obstacle in one study [19].

#### Female individual factors

Women constitute a barrier to male PMTCT involvement by not informing/involving their partners in ANC/PMTCT [16,25,35,40]. This aspect was identified in 17% of the studies. The reluctance on the part of women to involve their male spouses was grounded in numerous fears. There was the fear of divorce, accusations of infidelity or of bringing the infection into the relationship [35,40]. There was also the fear of stigmatization in case the woman was HIV-positive [35]. Finally the fear of experiencing domestic violence prevented women from bringing their male partners for counseling and testing [16].

#### Relationship dynamics

Weaker relationships either because couples are not cohabiting together or do not share affection with each other, constituted a barrier to male involvement [15,19]. This was probably linked to a lack of communication or a general demotivation of the men in the women’s affairs including ANC.

Fidelity within a relationship was also identified as potential barrier in that men who were faithful to their spouses were less likely to be involved in VCT and ANC [40,44], probably based on the general belief their own fidelity meant that their spouses were equally faithful and uninfected.

#### Disagreement with PMTCT teachings

Among the less prevalent factors, some men believe that PMTCT encourages the use of condoms within married couples [39]. Their disagreement with such PMTCT recommendations prevented male involvement.

There is also some impression from the men that prenatal HIV testing for pregnant women as recommended by the PMTCT programmes was infact a late event. Their belief was that the couple should have been given the chance to test before, and would have had the opportunity to consider whether or not to engage in child bearing in the first place [40]. Consequently for them this prenatal HIV testing was pointless.

### Facilitators of male involvement

Similar to barriers, facilitators of male PMTCT involvement were equally numerous and recurrent amongst studies in different settings. The different facilitators were grouped into four themes namely: health system facilitators, male individual facilitators, female individual facilitators and relationship dynamics facilitators. As with barriers, the relative importance of each facilitator was based on the number of studies reporting it. This criterion was used to rank the different facilitators as reported in Table 3.

**Table 3:** Summary table of facilitators of male PMTCT involvement

**FACILITATORS TO MALE PMTCT INVOLVEMENT**	
**Health system facilitators**	
**• **Invitation letters from health service inviting men to PMTCT [20,38,41,47,49].	
**• **Offering routine voluntary couple counselling [25,37,40,42].	
**• **Provision of counselling services during non-working hours [40,47].	
**• **Offering of counselling and testing for HIV at sites other than antenatal care [41,47].	
**• **Availability of health personnel to encourage testing and facilitate disclosure [19].	
**• **Change from voluntary counselling and testing to routine counselling and testing [34].	
**• **Offering of counselling and testing for HIV within antenatal settings [47].	
**• **Differential targeting and offering of counselling and testing of HIV to men accompanying their wives to the delivery wards [23].	
**• **Holding of open discussions on free prenatal HIV testing for partners [35].	
**• **Differential counselling for HIV positive women [32].	
**• **Community sensitization activities [20].	
**• **Availability of anti-retroviral drugs in the health centre [20].	
**Relationship dynamics factors**	
**• **Monogamous marriage or cohabitation of partners [14;25;42].	
**• **Discussion of PMTCT within the couple [14,48].	
**• **Sero-concordance for HIV [42].	
**Male individual facilitators**	
**• **Previous male testing for HIV [14,44].	
**• **Providing men with time to consider PMTCT recommendations [33,37].	
**• **Increased male knowledge concerning HIV and perceived benefits of PMTCT [19,48].	
**Female individual factors**	
**• **Lack of financial dependence on the part of women [15].	
**• **Positive attitudes of women towards disclosure of their test results [46].	

PMTCT=Prevention of mother-to-child transmission, HIV=Human Immuno-deficiency Virus.

#### Health system facilitators

Most of the activities which facilitated male involvement in ANC/PMTCT were linked to health systems interventions.

Sending out of invitation letters from the health centres inviting men to participate in ANC/PMTCT through their spouses was identified as a facilitator by a majority of the studies (21%) [20,36,39,45,49]. In one study, invitation letters were given to spouses by influential agents within the community [49]. The use of invitation letters was popular as it was perceived as a medical prescription which obliged the spouses to attend. Inviting male partners therefore appeared to be the most important facilitator.

Offering routine couple voluntary counselling and testing for HIV as the entry point into PMTCT services was identified in 17% of the studies [25,38,40,48]. Couple VCT serving as a standard of care may have eliminated stigma faced by men attending ANC. This relatively high proportion depicts the importance of this facilitator.

Providing ANC services for couples such as couple voluntary counseling and testing during weekends or non-working hours facilitated male partner involvement in ANC/PMTCT activities [38,45]. This was reported by 8% of the studies.

The site where voluntary counseling and testing (VCT) for HIV was offered to men was also identified by 8% of the studies [39,45]. There was a general agreement that a conducive site was needed. In some settings the offering of VCT for men at sites other than the overcrowded health centres [39,45] such as bars, churches [39] or even their jobsites, was a major facilitator to male partner involvement. This facilitator avoided the discomfort felt by men when attending the ‘female oriented’ ANC settings, and was a major boost to their involvement.

Among the less prevalent facilitators, the presence of a health personnel within the clinic whose role was to facilitate disclosure of HIV status amongst partners was reported in one study [19]. The assurance of the presence of such a person motivated men to attend and participate in PMTCT.

One study reported that change from voluntary counselling and testing to routine counselling and testing in accordance with WHO opt-out approach led to an increase in male partner HIV testing [34].

Even though probably almost in direct contrast with the fourth facilitator above, the main facilitator for the men’s PMTCT involvement reported by Katz et al. [47] was the offering and testing for HIV for men within the antenatal care settings. VCT in these settings was shown to increase the likelihood sharing of results and subsequent male PMTCT participation because of the greater investment in the partnership and interest in the child’s health [47].

A further facilitator of male involvement was the differential targeting and offering of VCT for HIV to the men who accompanied their wives to the delivery ward [23]. These men were usually more motivated in the health of their wives and future babies. Targeting them served as an ideal starting point for increasing male involvement in ANC/PMTCT [23].

Another facilitator was open discussions on free prenatal HIV testing for partners [35]. Such discussions by health personnel could have helped dissipate cultural myths and provided men with the knowledge of possible benefits of their involvement and where to seek services.

Differential counselling of women was also identified as a facilitator of male involvement. In these settings, HIV positive women were specifically required to bring along their spouses [32] for counselling and testing. This measure facilitated the adherence to PMTCT recommendations by selectively targeting the concerned male population.

#### Relationship dynamics facilitators

Certain aspects within the partnership facilitated male partner involvement in ANC/PMTCT.

Monogamous marriages or the cohabitation of partners were recognized facilitators reported by 13% of the studies [14,25,40]. This factor highlights the importance of stability within relationship, better quality of the relationships, and the role of dialogue and possibly mutual respect.

Partners discussing PMTCT was another facilitator of male involvement mentioned by 8% of the studies [14,46], and this was independent of marital/cohabitation status. This discussion permitted decision making within the couple and the dual implication of the couple in ANC/PMTCT.

Though not a desirable scenario, sero-concordance for HIV infection was a facilitator for increased condom use within the couple [42], a PMTCT recommendation. The reasons for this could be very diverse.

#### Male individual facilitators

Three major facilitators—history of previous male testing, time and knowledge concerning HIV and PMTCT—were each mentioned by 8% of the studies, were identified in this category.

Previous male testing for HIV was shown to facilitate male adherence to ANC/PMTCT recommendations [14,42]. This previous testing made men more knowledgeable about HIV, made them more implicated in their families health and enabled them to better support their spouses to prevent child HIV infection [14].

An important yet probably often neglected facilitator of male involvement was time. Men needed time to consider PMTCT recommendations and to get ready with their partners before getting involved [33,48]. Leaving room for men to take their time and make decisions facilitated the process of male involvement and improved adherence to PMTCT recommendations.

The knowledge of the male individual in question concerning HIV and PMTCT equally facilitated their involvement [19,46]. Where men were knowledgeable that HIV testing was beneficial for the baby, and that necessary treatment could be obtained, male involvement was increased [46]. Moreover, men’s knowledge on the perceived benefit of PMTCT, the perceived benefit from support and knowledge sharing among male peers as well as guidance from adults not to rush into violence or divorce because of HIV, were individual factors which facilitated male involvement in PMTCT [19].

#### Female individual factors

Female related facilitators were less prevalent as they were each mentioned by isolated studies.

The lack of financial independence on the part of women was a recognised facilitator of male involvement [15]. Financial dependence on the part of these women meant spouses were an indispensable source of income. This therefore increased male information towards ANC/PMTCT by their spouses with an overall increased male involvement and better adherence to PMTCT recommendations by the women in question [15].

Independent of finances, the attitudes of women towards disclosure of their HIV test results was a major gateway for male PMTCT involvement. Hence by sharing their test results with their partners women facilitated the entry of men into PMTCT [46] especially if testing was not done simultaneously.

## Discussion

The above results demonstrate that cultural factors, availability of HIV-related information and health system factors constitute the main barriers to male involvement in PMTCT activities. Specifically the most pertinent barriers noted include: the cultural perception of antenatal care as a woman’s place; the societal norm that men should not participate in ANC/PMTCT; the societal ridicule of men participating in ANC/PMTCT; the reluctance of men to learn their HIV status; the misconception by men that their partner’s HIV status is a proxy of theirs; the unawareness of ANC PMTCT services; time conflicts with ANC/PMTCT; long waiting times at the clinics; and the male unfriendliness of ANC/PMTCT services. Accordingly, many of the actions shown to facilitate male involvement in PMTCT have taken place at these levels. The most notable amongst these include: inviting men to ANC/PMTCT; offering routine voluntary couple counselling, the provision of services during non-working hours; offering HIV counselling and testing at other locations; community sensitization activities; open discussions for free prenatal HIV testing for partners; increased male knowledge concerning HIV and the perceived benefits of PMTCT.

The blend between minimizing these barriers and optimizing facilitators in a context-specific manner would help optimize the benefits of male involvement in PMTCT. The results imply a need not only for amendments but also context-specific adaptations if we are to achieve the public health benefits of male PMTCT involvement.

At the level of the health system these findings imply there is a need for reinforcement of the strategies used, if any, to improve male involvement in PMTCT. There is a need to actively invite and involve men in PMTCT activities through different means. These efforts of engaging men should consider the health and other needs of men rather than simply portray them as tools for women’s or infant’s health outcomes [4].

Furthermore, health service changes rendering ANC and PMTCT services more male-friendly [32] are necessary. These could include the implementation of couple antenatal counselling and testing as a routine within the health service [19,24,47], the reorientation of services towards both sexes [19], the possibility of couple/individual testing [48], the strengthening of couple counselling outside routine antenatal care [19], and the creation of male-friendly spaces within the ANC premises [39] amongst others.

Also within the health system, capacity reinforcement and motivation of the health service providers could improve the quality of services and minimize long waiting times within antenatal care [44,45]. Most often than not, resources are usually in short supply and antenatal clinics especially in developing countries are usually inadequately staffed [5]. Efforts need to be made to target the best use of available resources. One option may be the differential counselling for HIV infected women to bring along their husbands [32]. Another may be the selective counselling of men presenting at delivery wards [23]. Irrespective of the methods chosen, such selective measures may not only minimize time wastages, but may also ensure that limited resources and manpower are concentrated upon the population most likely to benefit from the interventions.

Still within the health system, the offering of VCT during non-working hours has been identified as a means to improve male uptake of PMTCT interventions [38,45]. The use of alternative but acceptable HIV testing sites was also suggested [39]. The implication of these suggestions within the health system is the careful consideration of the cost-effectiveness and acceptability of any interventions before implementation.

Cultural barriers represent the most pertinent barriers to male involvement in PMTCT. This is a multi-faceted barrier, the strongest elements being the recognition of antenatal care as the woman’s place P[19,23,38-41,43,44,48], and the associated discomfort for all men found within these settings [19,32,44]. Other cultural barriers focused on the power dynamics within the home [39,44]. A possible way forward may be to initiate diverse community-based initiatives to address underlying gender norms and societal attitudes towards male involvement in PMTCT activities. Men could be brought to realize it is unacceptable to preserve outdated cultural norms at the risk of losing their lives [11], and endangering infants. In so doing, we must however recognise the values that every society places in its culture and heritage. It is therefore important to work with community leaders to build upon and expand the present cultural expectations, such that additional social support is provided to women, and men be encouraged to participate in ANC/PMTCT activities [44]. Hence community-based programs that would normalize male ANC/PMTCT participation and minimize associated stigma need to be initiated.

The implications of our results for policy makers are numerous. The gap between global health policies and local realities must be addressed. The problem of finances is a recurrent problem, and the fear of the burden of care is enough to dissuade men from ANC/PMTCT interventions. Therefore, addressing health system financing mechanisms is a major step towards encouraging couples to seek care. Furthermore, the lack of concern for the local context of infant feeding has been identified as a limitation to the success of PMTCT programs in sub-Saharan Africa [39]. The policy implications of these findings should include universal free HIV testing [44], and free access to HIV care within the health service [35]. Also, whenever possible artificial milk should be provided freely to HIV-infected mothers who opt not to breastfeed.

Policy on the general organization of health services need to be addressed. Establishment of a widely disseminated health policy on HIV testing could minimize the fear of HIV testing [37]. A way forward could be to strengthen the policy on routine HIV testing in antenatal care to incorporate partners. Other measures could include the provision of pre and post-test counselling services for HIV on the same day [33], the availability of rapid HIV-testing at the clinic and increased confidentiality at every level of the ANC/PMTCT program.

Policies directly addressing PMTCT may also need to be addressed. Aspects such as the integration of domestic violence screening into the PMTCT programmes should be considered in order to minimize this obstacle within the community [16]. Furthermore, PMTCT programs should consider granting women the possibility to test more than once as this may provide multiple opportunities for getting partners involved [24]. Moreover, PMTCT models should also consider integrating discussion of finances within the family [40]. Family health budgets should have provisions for male care.

The lack of knowledge about HIV and the importance of male involvement in PMTCT have direct implications for information, education and communication initiatives. It highlights the need to increase male education on HIV/PMTCT and target information for men by various means [20,41,44]. Examples of these sensitization activities include the pasting of flyers and posters in areas frequented by men, and use of the media to discuss and encourage male participation in HIV/PMTCT [20]. The knowledge barrier also calls for increased training of health educators and the revision of educational messages provided by health counselors so far. Hence, counselling messages within ANC/PMTCT services should address spousal communication regarding sexual risks [42]. It should also encourage women to discuss VCT with their spouses before testing [25], and help them to elaborate plans to involve their partners early in PMTCT [40]. The message that a woman’s HIV status is not a proxy measure of that of her partner should be emphasized.

This review has some limitations. The major limitation is that only English reports were considered. Our findings are also limited to the databases we searched and may not provide a complete picture of the barriers and facilitators to male partner participation in PMTCT. Furthermore, most of the studies included in this review were from resource limited settings hence these results may not be applicable in higher resource settings. In addition, most of the included studies were conducted in urban settings in resource limited areas, thus limiting the applicability of some of these findings in rural settings.

## Conclusions

Optimal uptake and adherence to PMTCT recommendations is difficult for women whose partners are not supportive of their involvement [5]. Numerous facilitators and barriers exist to this male involvement. All the stakeholders in the control of HIV, especially PMTCT should endeavor to employ context specific strategies that limit the barriers and enhance the facilitators to male partner involvement. For these strategies to be feasible at a local level, it is important for health workers to identify the couples that need assistance the most. Further research using randomized controlled trials is needed to better understand the most effective strategies for enhancing male partner involvement in PMTCT. This would also require development of a tool for measuring male partner involvement in PMTCT.

## Competing interests

The authors declare none.

## Authors’ contributions

FM, LT and LM jointly conceived the research idea. CR and AW performed the study searches. FM and LM performed the data abstraction. FM made the first draft. All authors reviewed several versions of the manuscript. All authors read and approved the final manuscript.
